# Sodium Tanshinone IIA Sulfonate Prevents Radiation-Induced Toxicity in H9c2 Cardiomyocytes

**DOI:** 10.1155/2017/4537974

**Published:** 2017-03-12

**Authors:** Wenjing Zhang, Yi Li, Rui Li, Yaya Wang, Mengwen Zhu, Bowen Wang, Yanling Li, Dongyun Li, Ping Xie, Bin Liu

**Affiliations:** ^1^School of Clinical Medicine, Gansu University of Traditional Chinese Medicine, Lanzhou, Gansu 730000, China; ^2^The Institute of Medical Genetics, School of Basic Medical Sciences, Lanzhou University, Lanzhou 730000, China; ^3^Gansu Cardiovascular Institute, Lanzhou 730050, China; ^4^School of Stomatology, Lanzhou University, Lanzhou, Gansu 730000, China; ^5^School of Clinical Medicine, Ningxia Medical University, Yinchuan, Ningxia Hui Autonomous Region 750000, China; ^6^Department of Cardiology, Gansu Provincial Hospital, Lanzhou, Gansu 730000, China; ^7^Radiology Department, Gansu Provincial Hospital, Lanzhou, Gansu 730000, China

## Abstract

The present study was designed to elucidate the key parameters associated with X-ray radiation induced oxidative stress and the effects of STS on X-ray-induced toxicity in H9c2 cardiomyocytes. Cytotoxicity of STS and radiation was assessed by MTT. Antioxidant activity was evaluated by SOD and MDA. Apoptosis was measured by the flow cytometry, Hoechst 33258, clonogenic survival assay, and western blot. It was found that the cell viability of H9c2 cells exposed to X-ray radiation was significantly decreased in a dose-dependent manner and was associated with cell cycle arrest at the G0/G1 phase as well as apoptosis. STS treatment significantly reversed the morphological changes, attenuated radiation-induced apoptosis, and improved the antioxidant activity in the H9c2 cells. STS significantly increased the Bcl-2 and Bcl-2/Bax levels and decreased the Bax and caspase-3 levels, compared with the cells treated with radiation alone. STS treatment also resulted in a significant increase in p38-MAPK activation. STS could protect the cells from X-ray-induced cell cycle arrest, oxidative stress, and apoptosis. Therefore, we suggest the STS could be useful for the treatment of radiation-induced cardiovascular injury.

## 1. Introduction

Advances in the treatment and diagnosis of cancer and cardiovascular diseases have resulted in a great improvement in patient survival, but the growing population of long-term survivors has been compromised by the occurrence of comorbidities and complications throughout their lifespan, which can substantially affect their quality of life [1]. These chronic disorders include secondary malignant neoplasms, endocrine disorders, cardiopulmonary dysfunction, cardiovascular complications, and cognitive and psychosocial problems [2, 3], with cardiac injury being one of the most difficult complications to manage in the clinic [4]. For instance, patients receiving radiation therapy are often at a great risk for developing cardiomyopathy, which increases the risk for myocardial infarction and heart failure [5–7]. Moreover, the majority of cardiac catheterizations for congenital heart disease and some regular follow-up examinations, including chest X-rays, angiography, scintigraphy, and computed tomography scans, are often performed in children and adolescents who may experience intensive radiation exposure and are at a high risk for long-term adverse effects due to radiation [8–10]. Meanwhile, the use of medical ionizing radiation has been increasing dramatically in recent years, and cardiologists are also exposed to radiation from various medical sources, such as percutaneous coronary intervention, cardiac radiofrequency ablation, multidetector coronary angiography, and myocardial perfusion imaging scintigraphy [11].

Medical radiation sources are known to be cardiotoxic, but the underlying mechanism by which they induce cardiac damage is not fully understood [12]. Studies have demonstrated that radiation may generate oxygen free radicals, resulting in the generation of excessive reactive oxygen species that induce oxidative stress in cells and tissues [13]. The sustained generation of reactive oxygen species is a primary inducer of apoptosis [14]. Studies in mice have demonstrated that radiation causes acute injury in cardiomyocytes, leading to apoptosis [15]. The loss of myocytes causes thinning of the myocardium with a resulting increase in wall stress, which may also be linked to defective regenerative and adaptive pathways in the remaining myocardial cells [16]. These pathological changes ultimately result in congestive heart failure [17]. Accordingly, reducing oxidative stress is of great importance in preventing or suppressing apoptosis in order to protect the heart against radiation-induced injury [18]. The current treatment for radiation-induced heart disease mainly utilizes symptomatic and supportive approaches or surgery (cardiac transplantation); however, all these methods are largely ineffective. Therefore, the development of a new therapeutic approach is an urgent unmet medical need.

There is an increasing interest in the discovery and development of novel natural product-based approaches for the prevention and treatment of radiation-induced heart diseases. For instance, recent studies [19, 20] have revealed that sodium tanshinone IIA sulfonate (STS), a derivative of tanshinone IIA, elicits protective effects on cardiomyocytes against oxidative stress-mediated apoptosis. This plant is a well-known traditional Chinese medicine used for the treatment of various cardiovascular diseases, whose effects on the cardiovascular system are mediated by improving microcirculatory, vasodilatory, anticoagulant, antithrombotic, anti-inflammatory, free radical scavenging, and mitochondria-protective effects [21].

Tanshinone IIA is extracted from the roots of* Salvia miltiorrhiza*, and, as an active diterpenoid component, it can prevent or slow the progression of cardiovascular diseases [22]. STS also has been used clinically as an approved drug by the State Food and Drug Administration of China [23, 24]. Its cardioprotective effects might be mediated by free radical scavenging properties and antioxidant activities [25]. There is increasing evidence supporting that STS relies on such an action to attenuate radiation-induced pulmonary and renal diseases [26]. Therefore, we hypothesized that STS could have preventive effects on radiation-induced heart disease. To this end, in the present study, we characterized the effects of STS against radiation-induced oxidative stress and the possible underlying mechanisms using H9c2 cardiomyocytes as an in vitro cell-based model. It was hoped that the results from the present study would provide a basis for the future development of STS as an anti-radiation-induced heart disease reagent in preclinical and clinical settings.

## 2. Materials and Methods

### 2.1. Chemicals

STS was obtained from Shanghai First Chemical Company (Shanghai, China). Dulbecco's modified Eagle medium (DMEM)/F12 medium was purchased from Gibco (Grand Island, NY, USA). Fetal bovine serum was from Invitrogen (Carlsbad, CA, USA). Hoechst 33258 was obtained from Invitrogen (Eugene, OR, USA). 3-(4,5-Dimethyl-2-thiazolyl)-2,5-diphenyl tetrazolium bromide (MTT), dimethyl sulfoxide (DMSO), and Giemsa were from Sigma (Sigma-Aldrich, St. Louis, MO, USA). The lactate dehydrogenase (LDH), superoxide dismutase (SOD), and malondialdehyde (MDA) assay kits were obtained from Nanjing Jiancheng Bioengineering Institute (Nanjing, China). Rabbit polyclonal antibodies for p38, phosphorylated p38, ERK, and phosphorylated ERK were purchased from Cell Signaling Technology (Beverly, MA, USA).

### 2.2. Cell Culture

H9c2 cardiomyocytes, a subclone of the original cell line derived from embryonic BD1X rat heart tissue, were obtained from the Chinese Academy of Sciences Cell Bank (Shanghai, China) and cultured in DMEM/F12 medium containing 10% (v/v) fetal bovine serum, penicillin (100 U/mL), and streptomycin (100 *μ*g/mL). The cells were maintained at 37°C in a humidified atmosphere with 5% CO_2_. The medium was replaced every 2-3 days, and the cells were passaged as they grew to 80% confluence; they were subcultured or subjected to subsequent experiments. The purity of STS used in the present study was >95%, and STS was dissolved in DMEM/F12 medium. The cells were divided into the following groups: 0 Gy (control) group; 2-Gy radiation (2 Gy) group; 4-Gy radiation (4 Gy) group; 6-Gy radiation (6 Gy) group; 8-Gy radiation (8 Gy) group; 10 *μ*g/mL STS (STS) group; 2 Gy + 10 *μ*g/mL STS (2STS) group; 4 Gy + 10 *μ*g/mL STS (4STS) group; 6 Gy + 10 *μ*g/mL STS (6STS) group; and 8 Gy + 10 *μ*g/mL STS (8STS) group.

### 2.3. Irradiation Procedure

The cells were seeded with fresh culture medium and cultured for 24 h prior to the radiation treatment. A single dose of 2, 4, 6, or 8 Gy of X-rays was administered to the cells using a Siemens PRIMUS high energy linear accelerator (Siemens AG, Erlangen, Germany), operating at a dose-pulse rate of 6 MV/min with a source-to-H9c2 cell distance of 100 cm. After radiation, the cells were maintained at 37°C and 5% CO_2_, and the culture medium was replaced every 24 h.

### 2.4. 3-(4,5-Dimethyl-2-thiazolyl)-2,5-diphenyl Tetrazolium Bromide (MTT) Assay

To determine the cytotoxicity of STS, H9c2 cells were seeded at 10^5^ cells/mL in 96-well plates. The experiments were carried out after cell exposure to serum-free medium for at least 16 h, and the cells reached more than 80% confluence on the plate bottom. After discarding the old medium, the cells were pretreated with varying concentrations of STS for 24 h, 48 h, and 72 h, respectively. MTT was dissolved in phosphate-buffered saline (PBS) and added to each well at a final concentration of 5 mg/mL. The cells were then incubated at 37°C for 4 h. The medium was discarded, and 150 *μ*L of dimethyl sulfoxide was added to each well. The absorbance of each well at 570 nm was quantified using a spectrophotometer (Bio-Rad, Philadelphia, PA, USA).

Cell viability was determined by the MTT assay. In brief, the cells were plated onto 96-well tissue culture plates (cell density, 4 × 10^3^ cells/well). STS was added to the medium at the doses indicated for 24 h, followed by exposure to X-ray radiation, and then cultured for another 24 h. Cell viability was then monitored. Each treatment and control was assayed in at least six wells, and the cell viability was expressed as a percentage of that of the control cells.

### 2.5. LDH Release Assay

Following pretreatment with STS for 24 h, the cardiomyocytes were irradiated with 2, 4, 6, or 8 Gy of X-rays. The culture medium was collected at 24 h after X-ray radiation, and the LDH activity was measured by means of a colorimetric assay using a commercial LDH assay kit, according to the manufacturer's instructions.

### 2.6. Antioxidant and Lipid Peroxide Assay

The MDA content was measured as an end product of lipid peroxidation. The defense systems against free radical attack were assessed by the measurement of the activities of SOD. The H9c2 cardiomyocytes were pretreated with or without STS for 24 h before cell exposure to different doses (2, 4, 6, and 8 Gy) of X-ray radiation, and they were cultured for another 24 h. Then, they were washed once with ice-cold PBS and lysed in ice-cold RIPA lysis buffer containing 1 mM phenylmethylsulfonyl fluoride and phosphatase inhibitor for 30 min. After centrifuging the lysates at 12,000 rpm and 4°C for 10 min, the supernatants were collected for measurements of MDA production and SOD activity. The activities of SOD and catalase as well as the contents of MDA were analyzed in H9c2 cardiomyocytes with or without STS pretreatment and exposure to X-ray radiation, according to the manufacturer's protocol (Nanjing Jiancheng Bioengineering Institute).

### 2.7. Cell Morphology

The cells were seeded on coverslips, exposed to radiation, and incubated in the presence or absence of STS for 48 h under the same conditions described above. The culture medium was removed, Hoechst 33258 was added to each well, and the cells were incubated for an additional 10 min. The coverslips were then washed three times with PBS, placed onto glass slides, and covered with mounting medium. Next, the cells were harvested at the indicated times and washed twice with cold PBS. The nuclear fluorescence was visualized under a Zeiss Axioplan microscope (Zeiss, Germany).

### 2.8. Clonogenic Assay

A clonogenic assay was performed to determine the effects of STS treatment on the survival and division of the radiation-treated H9c2 cells. Briefly, the cells were seeded into 60-mm dishes at a cell density of 1 × 10^3^ cells/well in triplicate, pretreated with STS for 24 h, and then irradiated at different doses (0–8 Gy) using an X-ray irradiator operating at a dose-pulse rate of 6 MV/min. At 24 h after the radiation treatment, the culture medium was replaced with fresh complete medium containing STS, and the cells were incubated at 37°C for 8–12 days to allow for colony formation. The colonies were fixed in methanol for 20 min and stained with 0.5% Giemsa for 30 min. The number of colonies (≥50 cells) was scored under a microscope. The surviving fraction was calculated as the ratio of the plating efficiency of the treated cells to that of the control cells.

### 2.9. Apoptosis Assay

The effects of STS on apoptosis were determined using the Annexin V/PI double-staining method. After exposure to X-ray radiation for 24 h, the H9c2 cardiomyocytes pretreated with STS or control were centrifuged to remove the medium. Next, the cell pellet was washed with PBS and stained with Annexin V in binding buffer (10 mM HEPES, 140 mM NaCl, and 2.5 mM CaCl_2_) for 20 min. At 10 min before the end of incubation, PI was added to this cell suspension in order to stain necrotic cells, according to the manufacturer's instructions (BioVision, Inc., Palo Alto, CA, USA). The cells were analyzed with a FACSCAN flow cytometer (Becton Dickenson Biosciences, San Jose, CA, USA), and the stained cells in the FL1-H and FL2-H channels were analyzed.

### 2.10. Cell Cycle Detection

The H9c2 cells in the logarithmic growth phase were harvested and seeded in DMEM containing 10% FBS at a density of 2 × 10^6^ cells per flask. After allowing the cells to adhere, the culture medium was removed and replaced with medium containing STS or control, and the cells were cultured for 24 h before radiation. After X-ray radiation at different doses, the cells were cultured for 24 h, harvested, washed once with cold PBS, resuspended in 1 mL of PBS, and then fixed with 2 mL of dehydrated ethanol for 30 min. The cells were collected, washed once with PBS, and then stained with 50 g/mL PI at room temperature in the dark for 30 min. The cells were then analyzed by flow cytometry (Coulter XL, Beckman Coulter, Inc., Fullerton, CA, USA) to determine the cell cycle distribution.

### 2.11. Western Blotting Analysis

After treatment with STS or control, the cells at 80% confluence were collected and washed three times with PBS. Radioimmunoprecipitation assay buffer was added to extract the total protein. Equal amounts of protein from the samples and controls were loaded onto 12% sodium dodecyl sulfate-polyacrylamide gels for protein separation. The proteins were then transferred to nitrocellulose membranes, and the membranes were blocked with 5% nonfat dried milk for 3 h. The membranes were then incubated with primary antibodies (p38 at 1 : 1000, p-p38 at 1 : 1000, Bcl-2 at 1 : 1000, Bax at 1 : 1000, and cleaved caspase 3 or caspase-3 at 1 : 1000) overnight at 4°C. After being washed with Tris-buffered saline (pH 7.2) containing 0.05% Tween 20 (TBST), the membranes were incubated with the secondary antibody at room temperature for 1.5 h. Finally, the membranes were washed with TBST and incubated with the enhanced chemiluminescence reagent to detect the proteins of interest. The levels of GADPH were used as loading controls.

### 2.12. Statistical Analysis

Statistical analyses of the results from the present study were performed using SPSS17.0 (version 12.0; IBM, USA). The results were expressed as means ± standard deviation (SD). Comparisons between the various levels of radiation with or without STS treatment were conducted by two-way analysis of variance (ANOVA); a paired *t*-test was used to evaluate differences between the same levels of radiation versus without treatment; comparisons between groups were performed using one-way ANOVA. A level of *p* < 0.05 was considered statistically significant.

## 3. Results

### 3.1. Radiation Induces Cell Growth Inhibition and Apoptosis in H9c2 Cells in a Dose-Dependent Manner

**Figure 1:**
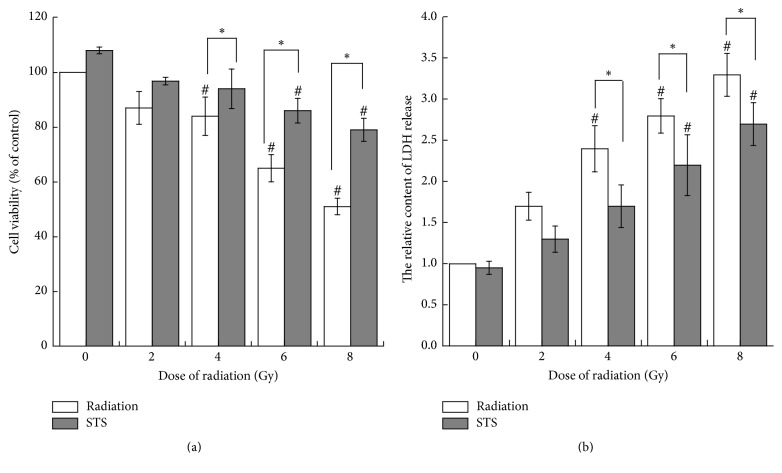
STS attenuated radiation-induced cytotoxic effects in H9c2 cells. (a) The effects of STS on cardiac cell viability after the cells were exposed to X-ray radiation, as measured by the MTT assay. (b) The effects of STS on X-ray radiation-induced LDH release in H9c2 cells. ^#^*p* < 0.05 versus the 0-Gy radiation group; ^*∗*^*p* < 0.05 STS treatment group versus the radiation alone group. All experiments were repeated three times.

The H9c2 cells were exposed to different doses of radiation (0, 2, 4, 6, and 8 Gy), and the MTT assay was performed to evaluate the cell viability. Radiation decreased the cell viability in a dose-dependent manner (Figure 1(a)). The cell viability in the X-ray-treated groups was significantly lower than that of the control (*p* < 0.05). Exposure to 6-Gy and 8-Gy radiation was associated with a greater loss of cell viability, compared with the 2-Gy and 4-Gy groups. LDH, which leaks from cells after plasma membrane disruption, can also be used as an indicator of cell injury. As shown in Figure 1(b), a marked increase in LDH activity was observed at 24 h after X-ray exposure.

**Figure 2:**
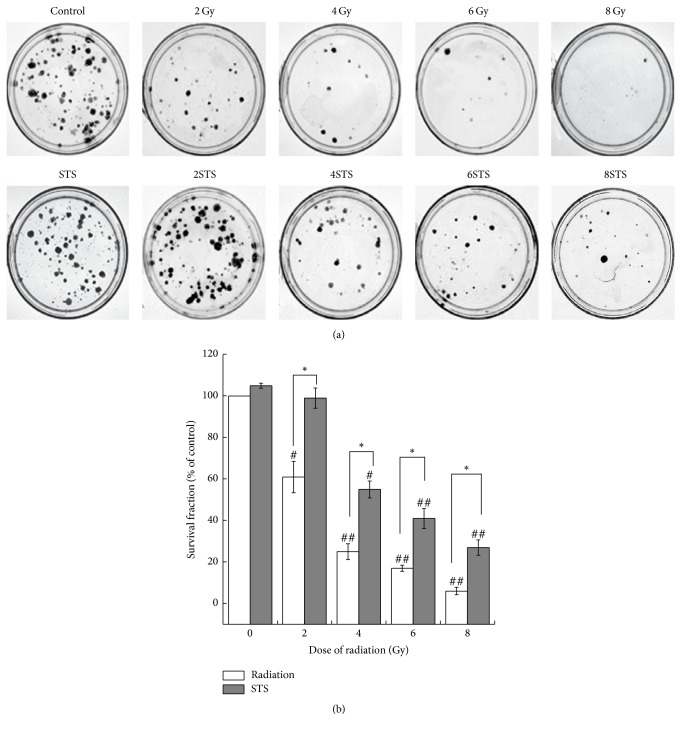
STS promoted cell proliferation activity in radiation-treated H9c2 cells. (a) Representative images of clonogenic assays show that radiation exposure inhibited the colony-forming capacity in a dose-dependent manner and that STS alleviated the suppressive effect of radiation. (b) The results of the clonogenic assays were normalized to the clonogenic survival of nonradiated control cells, and the survival fractions were plotted (*n* = 3). ^#^*p* < 0.05 and ^##^*p* < 0.01 versus the 0-Gy radiation group; ^*∗*^*p* < 0.05 versus the corresponding radiation alone groups.

**Figure 3:**
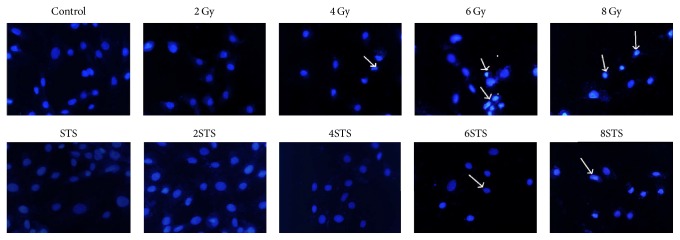
Hoechst 33258 staining was employed to examine apoptosis at 24 h after radiation. Radiation resulted in DNA condensation in H9c2 cells. Chromatin condensation, indicative of apoptosis, and apoptotic bodies were examined. Arrows indicate apoptotic nuclei and apoptotic bodies (10 × 20).

**Figure 4:**
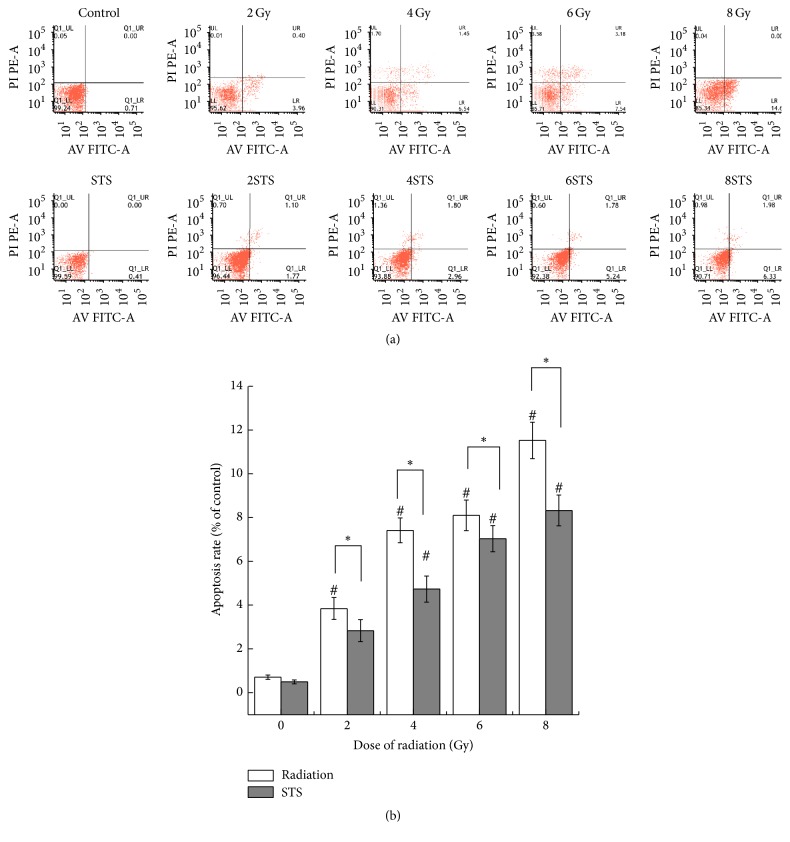
Effect of STS on the X-ray radiation-induced apoptosis in H9c2 cardiomyocytes. (a) Detection of apoptotic cells by Annexin V/PI double-staining. Cardiomyocytes were treated with or without STS, stained with Annexin V and PI, and analyzed by flow cytometry. (b) Quantitative analysis of apoptosis. The data are expressed as means ± SD (*n* = 9). ^#^*p* < 0.05 versus the control group; ^*∗*^*p* < 0.05 versus the corresponding radiation alone group.

In order to determine the mechanisms of the decreased cell viability after radiation, we analyzed the number of apoptotic cells, cell division, and cell cycle distribution after radiation. The observed cytotoxic effects were also confirmed with Hoechst 33258 staining, a clonogenic survival assay, and a flow cytometric assay. The cells exposed to various doses (2, 4, 6, and 8 Gy) of radiation showed a significantly reduced number of colonies (Figure 2(b)). The number of colonies in the 2-Gy and 4-Gy groups was markedly higher than that in the 6-Gy and 8-Gy groups. The results from the assays demonstrated that X-ray radiation significantly suppressed the growth of H9c2 cells in a dose-dependent manner, compared with the control. In addition, the higher X-ray radiation doses increased cell necrosis and apoptosis. As shown in Figure 3, normal morphology and nuclei were observed in the control group. Chromatin condensation, indicative of apoptosis, was shown by Hoechst staining in the radiation groups. Typical apoptotic cells with fragmented chromatin, chromatin condensation, or apoptotic bodies were observed in the 8-Gy group (Figure 3). As shown in Figure 4, the percentages of apoptotic cells in the radiation groups were significantly higher than that of the control group.

### 3.2. STS Ameliorates Radiation-Induced Apoptosis in H9c2 Cells

**Figure 5:**
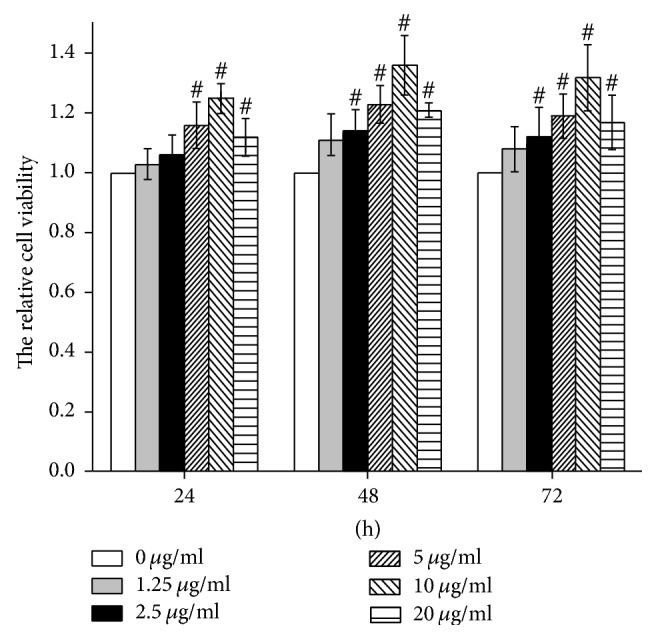
Effects of STS on cardiac cell viability as determined by the MTT assay. The cells were treated with various concentrations (1.25, 2.5, 5, 10, and 20 *μ*g/mL) of STS for 24, 48, and 72 h. The data are expressed as the mean ± SD. ^#^*p* < 0.05 versus the control group. All experiments were repeated three times.

To observe the effect of STS on cell viability and to select the proper STS concentration for subsequent assays, the H9c2 cells were exposed to various concentrations of STS (1.25–20 *μ*g/mL). The results of the MTT assays showed an increase in cell viability as the STS concentration increased from 1.25 to 10 *μ*g/mL. The proliferation rate was significantly increased when the cells were treated with 10 *μ*g/mL STS for 48 h (Figure 5). However, an inhibitory effect on cell proliferation was observed when the concentration of STS was greater than 20 *μ*g/mL. Although the mechanism for the high-dose-STS-induced cell growth inhibition was not clear, the STS concentration of 10 *μ*g/mL was selected for subsequent experiments.

The H9c2 cells were pretreated with STS (10 *μ*g/mL) for 24 h prior to exposure to different doses (2, 4, 6, and 8 Gy) of radiation. There was a significant difference in cell viability between the radiation alone group and the pretreatment with STS group. Compared with the same level of radiation group, the cell viability in the STS group was enhanced (Figure 1(a)). In addition, pretreatment with STS significantly decreased the LDH levels (Figure 1(b)). Next, clonogenic assays were performed to determine if STS treatment had any impact on radiation-induced H9c2 cell survival and division. Compared with the radiation groups, the number of colonies in the 2STS, 4STS, 6STS, and 8STS groups were 99 ± 4.9%, 55 ± 4.1%, 41 ± 4.8%, and 27 ± 3.7%, respectively, indicating that STS reduced the radiation-induced growth inhibition of H9c2 cells (Figure 2). STS treatment inhibited the apoptosis of the cultured cardiomyocytes. Hoechst 33258 staining demonstrated that the chromatin condensation and number of apoptotic bodies were decreased in the cells pretreated with STS (Figure 3). Similarly, STS treatment significantly decreased the percentages of apoptotic cells compared with the corresponding (2, 4, 6, and 8 Gy) radiation groups (Figure 4).

### 3.3. STS Exerts Antioxidant and Lipid Peroxidation Activities

**Figure 6:**
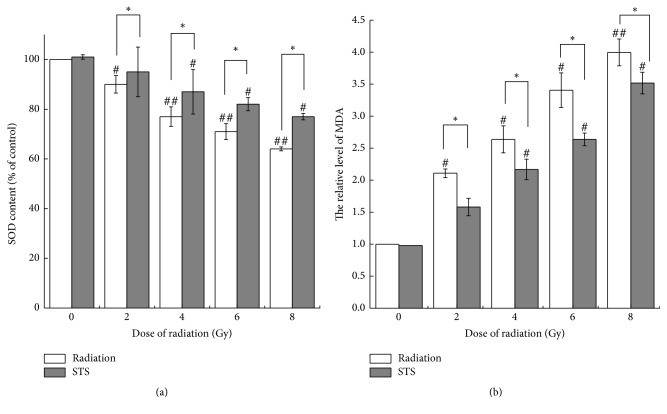
STS treatment ameliorated radiation-induced myocardial oxidative stress. Antioxidant activity was determined by measuring (a) superoxide dismutase (SOD) activity and (b) malondialdehyde (MDA). The results are expressed as the mean ± SD. ^#^*p* < 0.05 and ^##^*p* < 0.01 versus the control group; ^*∗*^*p* < 0.05 versus the corresponding radiation alone group. All experiments were repeated three times.

During radiation, severe oxidative stress leads to lipid peroxidation [27]. To explore the mechanism underlying the antioxidant activity of STS, we evaluated the activities of MDA and SOD; the activities of SOD in the radiation alone groups were significantly decreased, compared with the control group (Figure 6(a)), and the contents of MDA in the radiation alone groups were 2.11-, 2.64-, 3.41-, and 4-fold higher than that in the control group (Figure 6(b)). Pretreatment with STS remarkably reversed these alterations (Figure 6). STS treatment significantly downregulated the contents of MDA and enhanced the levels of SOD in cells exposed to X-ray radiation.

### 3.4. STS Reverses the Effects of Radiation on the Cell Cycle

**Figure 7:**
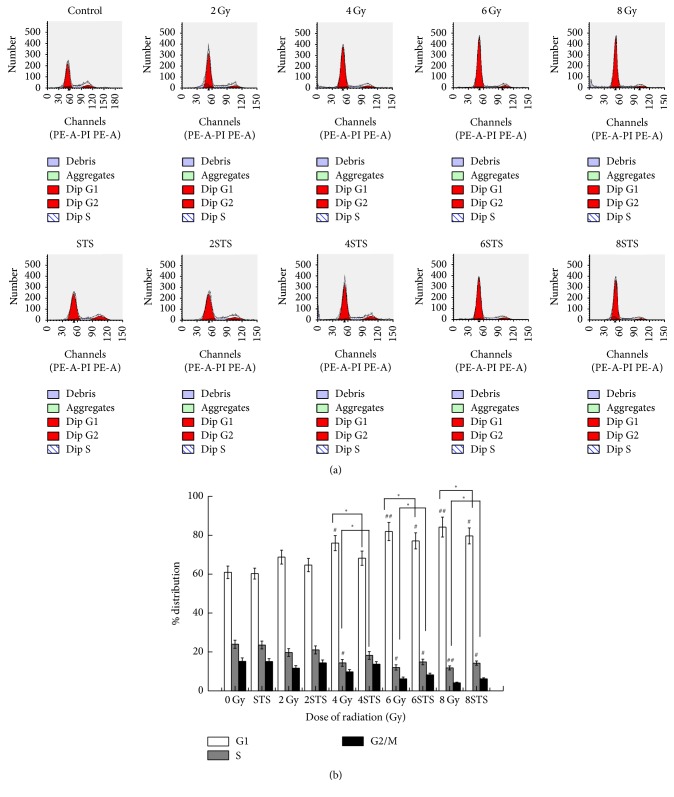
Radiation suppressed the growth of H9c2 cells via an apoptosis-dependent mechanism, and STS reversed it. PI staining and flow cytometric analyses were employed to examine apoptosis and the cell cycle distribution at 24 h after exposure to various doses of radiation (*n* = 3). (a) Representative flow cytometry graphs of the cell cycle analysis of cells irradiated with or without STS pretreatment. (b) The cell cycle analysis results of H9c2 cells are presented as the mean ± SD. ^#^*p* < 0.05 and ^##^*p* < 0.01 versus the control group; ^*∗*^*p* < 0.05 versus the corresponding radiation alone group.

The flow cytometric assay showed that, compared with the control, an increase in the population of cells at the G0/G1 phase in the radiation groups was seen after X-ray radiation in a dose-dependent manner, ranging from 60.9% to 84.14%, which was reversed by STS pretreatment (Figure 7). STS reduced G0/G1 cell cycle arrest.

Compared with the radiation groups, the percentages of cells at the G0/G1 phase were decreased (60.2%  ±  2.8% versus 64.6%  ±  3.4%, 68.12%  ±  3.65%, 77%  ±  4.12%, and 79.59%  ±  4.1%, resp., *p* < 0.05).

### 3.5. STS Modulates the Levels of Apoptosis-Related Proteins

**Figure 8:**
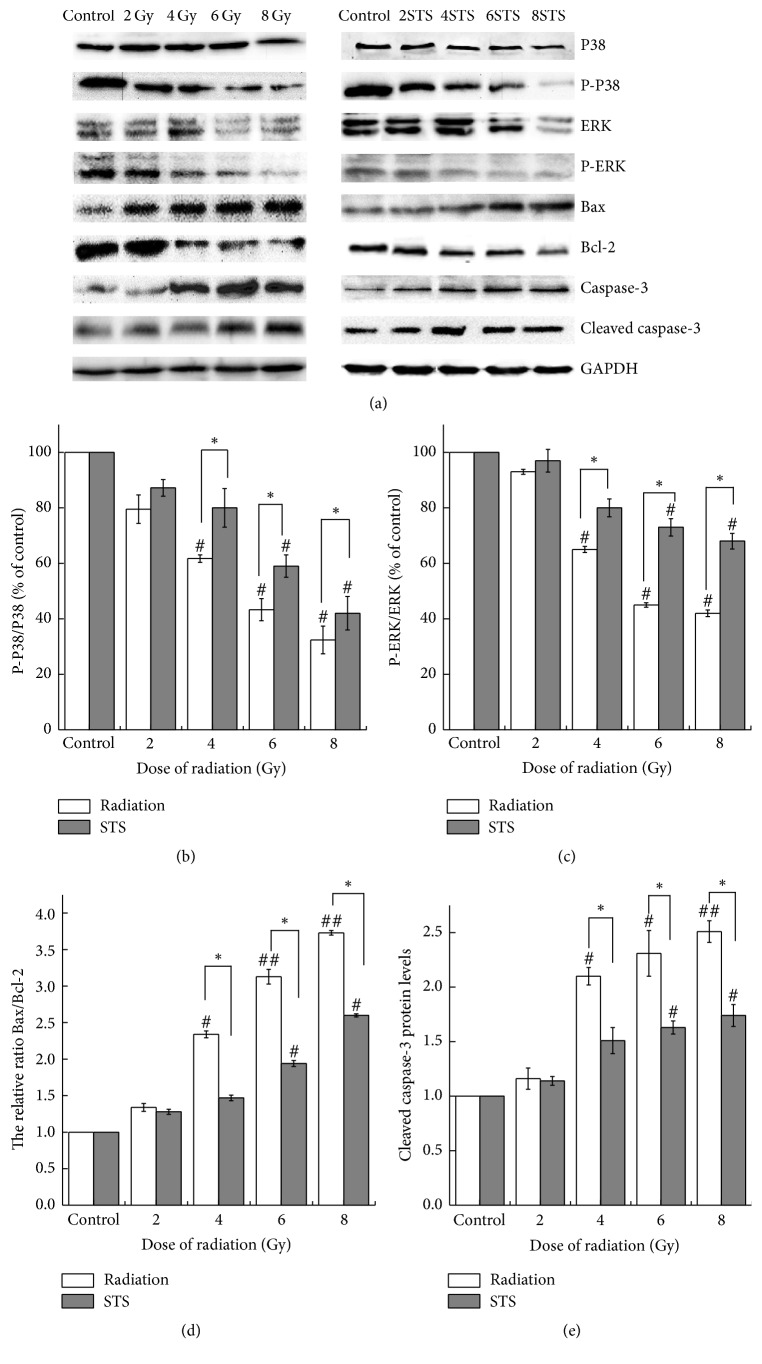
STS modulated the expression of Bcl-2, Bax, Bax/Bcl-2, p-p38, and p-ERK as well as the activation of caspase-3 in the radiation alone group (a), compared to the control group, as detected by western blotting. (b), (c), (d), and (e) represent the relative protein expression levels and are presented as the mean ± SD. ^#^*p* < 0.05 and ^##^*p* < 0.01 versus the control group; ^*∗*^*p* < 0.05 versus the corresponding radiation alone group. All experiments were repeated three times.

Compared with the control, the protein expression levels of the antiapoptotic gene Bcl-2 were markedly decreased, and the proapoptotic gene Bax was markedly increased; therefore, the Bax/Bcl-2 ratio was increased accordingly in the radiation groups; the expression levels of caspase-3 were also increased (Figure 8(e)) in the radiation group. These effects were prevented by pretreatment with STS: the expression level of Bcl-2 was increased; the expression levels of Bax as well as caspase-3 were decreased, and the Bax/Bcl-2 ratio was decreased (Figure 8).

To further assess the signaling pathways potentially involved in the process of cardiomyocyte apoptosis induced by radiation, we examined the key components of the MAPK pathways in the radiation alone groups. As shown in Figure 8, there was a marked decrease in p-ERK and p38-MAPK in the radiation groups, compared with the control group. STS pretreatment upregulated the expression levels of p-ERK and p38-MAPK.

## 4. Discussion

Radiation exposure from various medical sources, such as cardiac catheterizations for congenital heart disease and radiation therapy for cancer, is almost unavoidable and often causes irreversible cardiac injury [28] that is accompanied by increased oxidative stress. The overproduction of radicals leads to increased myocardial apoptosis, ultimately resulting in cardiac failure [29]. The severity of radiation-induced heart disease, in terms of the mortality and morbidity, has been reported previously [30]. However, the detailed mechanism underlying radiation-induced heart disease and its treatment and prevention strategies remain elusive [31].

In the present study, we first determined the cytotoxicity of radiation in H9c2 cardiomyocytes by assessing the cell viability, LDH leakage, clonogenicity, and apoptosis. X-ray radiation reduced the cell viability in a dose-dependent manner. The results obtained from the cell viability assay were consistent with the findings from the LDH assay. LDH leakage, as a marker of necrotic cellular death, was measured in the cell culture medium. There were significant differences in the LDH levels between the control group and the radiation alone groups. X-ray radiation-induced LDH release was dose-dependent. Compared with the control group, the X-ray-treated cells underwent apoptosis, as shown by Hoechst 33258 staining. Similar results were also obtained with a clonogenic assay, demonstrating that X-ray radiation promoted cardiomyocyte apoptosis and inhibited cell proliferation.

Given the central role of oxidative stress in the pathogenesis of radiation-induced heart disease, ameliorating oxidative stress through treatment with antioxidants might be an effective prevention strategy. STS has been utilized by traditional Chinese medicine practitioners for its antioxidant effects and has been shown to reduce the risk factors for several cardiovascular diseases [32]. The beneficial role of STS in improving radiation-induced heart disease, however, has not yet been demonstrated. In this study, we used a cell-based radiation-injury model to investigate the antioxidant effects of STS in order to demonstrate the possibility of using this agent for the prevention of radiation-induced cardiomyopathy. The MDA level indicates the membrane lipid peroxidation level during oxidative damage [33] and is indicative of cardiomyocyte oxidative damage, while the endogenous antioxidant SOD protects the cells against oxidative stress. In the present study, the treatment of cells with radiation increased the intracellular MDA levels and decreased the SOD levels, compared with the control cells, which were reversed by STS. STS treatment decreased the radiation-induced cell death, as measured by a clonogenic assay, and restored the alterations of H9c2 morphology and nuclear condensation to nearly normal levels. These results suggested that the antioxidant activity of STS might help protect the cells from radiation-induced cytotoxicity.

In the present study, the flow cytometric analysis results revealed that X-ray radiation alone significantly increased the percentages of necrotic and apoptotic cells, as compared with the control group; moreover, STS treatment significantly decreased the rate of apoptosis in cells exposed to radiation in a dose-dependent manner. STS significantly attenuated cell injury induced by radiation in H9c2 cardiomyocytes, suggesting that STS might be capable of protecting H9c2 cardiomyocytes against radiation-induced injury. The cardioprotective effect of STS was related to its ability to reduce myocardial cell apoptosis and damage induced by oxidative stress.

In the present study, cell cycle analysis indicated that radiation-induced damage increased the proportion of cells in the G0/G1 phase and reduced the proportion of cells in the S phase, demonstrating that X-ray radiation reduces cell survival by arresting cell cycle progression as well as inducing cell apoptosis. STS pretreatment increased the proportion of cells in the S phase. The number of S phase cells could reflect the ability of cells to proliferate, indicating that STS may inhibit radiation-induced cell cycle arrest in H9c2 cells.

Apoptosis occurs with Bax translocation to the mitochondria, which releases apoptotic factors during the apoptosis process [34]. Bcl-2 inhibits Bax activation, and caspase-3 is the effector molecule that is responsible for DNA fragmentation during the terminal events preceding cell death [35, 36]. Our results showed that X-ray exposure resulted in a decreased expression of Bcl-2, an increased expression of Bax and caspase-3, and an increased Bcl-2/Bax ratio. Compared with the radiation alone groups, the levels of Bcl-2 and Bcl-2/Bax increased while Bax and caspase-3 decreased markedly in the radiation + STS group, indicating that STS reduced myocardial cell apoptosis through modulating the expression of Bcl-2 family proteins.

p38-MAPK, the major member found in cardiac tissues, plays a key role in diverse biological processes with varied outcomes, either promoting cell death or ensuring survival. p38-MAPK is activated simultaneously with JNKs, which promote survival as in the case of cardiac myocytes exposed to oxidative stress [37]. The beneficial effects of p38 activation include an antihypertrophic action and enhanced myocardial adaptation to stress, perhaps through myocardial angiogenesis [38]. As far as ERKs are concerned, they are involved in regulation of the cell cycle, cell proliferation and differentiation, and cell migration as well as in the stress response. ERKs appear to function principally in favor of cell survival, exerting an antiapoptotic role [39]. In the present study, we also demonstrated that STS treatment activated the p38-MAPK pathway, which may be associated with its protective effect against radiation-induced injury in H9c2 cells. The expression of p-p38 was increased markedly, indicating that STS enhanced the survival of cells exposed to radiation, at least in part by promoting the p38-MAPK signaling pathways.

## 5. Conclusions

Our results from the present study demonstrated that STS protected myocytes from X-ray radiation-induced injury via suppressing apoptosis-related gene activation and preventing inappropriate apoptosis. These findings suggest that STS may be a promising agent for treating radiation-induced cardiomyocyte injury.
